# 

**Published:** 1905-06-03

**Authors:** 


### NOTICE TO CORRESPONDENTS.

All MSS., Letters, Books for Review, and other matters intended for the Editor should be addressed The Editor, "The Hospital," The Hospital Buildings, 28 & 29 Southampton Street, Strand, W.C.

The Editor cannot undertake to return rejected MS., even when accompanied by stamped directed envelope.

**Notice to Advertisers and Subscribers.**

All Advertisements, Orders for Copies of The Hospital, and Business Communications should be addressed to **THE MANAGER (Not to the Editor),** The Hospital, 28 & 29 Southampton Street, Strand, London, W.C.

The Cost of Subscription, post free, is at follows:—Per week, 3½d.; per quarter, 3s. 3d.; per half-year, 6s. 6d.; per annum, 13s. Foreign and Colonial:—Per annum, 19s. 6d., payable, in all cases, in advance.

**NOTICE.—Vol. XXXVII. of "The Hospital" October 1904 to March 1905), in handsome cloth binding, will shortly be on sale at the office of this Journal, price 10s. 6d. Cases for binding the Numbers contained in this Volume 2s. Index to Vol. XXXVII., 3½d., post free.**

**The Monthly Part for June is now ready. Price 1s., by post, 1s. 3d.**

### BOOKS RECEIVED.

Adams Bros.

"The Physiological Aspect of Dietary." By A. S. Wauchope-Watson.

Baillière, Tindall and Cox.

"Laryngeal Phthisis." By R. Lake, F.R.C.S.

"Diet and Disease." By J. Sim Wallace, M.D.

"Mucous Membranes: including Mucin and Malignan[illegible] By W. Stuart-Low, F.R.C.S.

John Bale, Sons, and Danielsson.

"Verb. Sap. on going to West Africa." By Alan Field, F.R.G.S.

"The Maintenance of Health in the Tropics." By W. J. Simpson, M.D.

### MEDICAL APPOINTMENTS.

**Arthur Alex. Blakiston**, M.R.C.S., L.S.A., Deputy Medical Officer for the Third District of the Wells (Somerset) Union. **E. Chittenden Bridges**, M.D., B.S.Durh., M.R.C.S.Eng., L.R.C.P.Lond., Honorary Anæsthetist to the Victoria Hospital for Children, Chelsea. **Joseph Cunning**, M.B., B.S.Melb., F.R.C.S. Eng., Assistant Surgeon to the Victoria Hospital for Children, Chelsea. **Charles Heaton**, M.D.Brux., M.R.C.S., L.R.C.P., Honorary Assistant Visiting Surgeon to the Royal Sea-bathing Hospital, Margate. **George Henry James Hooper**, M.D. Lond., L.R.C.P.Lond., M.R.C.S., J.P., Chairman of the Sutton (Surrey) District Council. **Miss E. Hooper**, M.B., B.S.Lond., Resident Clinical Assistant to the Hospital for Women and Children, Leeds. **P. Oswald Jollie**, L.R.C.P.&S.Edin., L.F.P.&S. Glasg., Medical Officer and Poor-law Vaccinator for Colmonell, Ayrshire. **John Lewis Lock**, M.A., M.B., B.C.Cantab., M.R.C.S., L.R.C.P., Medical Officer to the Infirmary of the Uxbridge Union. **H. Willoughby Lyle**, M.D., B.S.Lond., F.R.C.S.Eng., Ophthalmic Surgeon to the Royal Ear Hospital, Soho, and Consulting Ophthalmic Surgeon to the Beckenham Cottage Hospital. **Percy Sargent**, M.A., M.B., B.C.Cantab., F.R.C.S.Eng., Surgeon to Outpatients, Victoria Hospital for Children, Chelsea.

### BOOKS FOR

### Midwives and Maternity Nurses.

Published by THE SCIENTIFIC PRESS, LTD.

### A COMPLETE HANDBOOK OF MIDWIFERY.

By J. K. WATSON, M.D.

Designed to meet the requirements of the Central Midwives Board.

Containing over 350 pp., and Illustrated with 143 Diagrams. Cloth, 6/- net, 6/4 post free.

**HOW TO BECOME A CERTIFIED MIDWIFE.**

By E. Appel, M.B. 1/1 post free.

**PRELIMINARY LECTURES ON MIDWIFERY.**

By M. Caley. 1/8 post free.

**DISTRICT MIDWIFERY CASE-BOOK.**

By M. Caley. 7d. post free.

**THE MIDWIVES' POCKET-BOOK.**

By Honnor Morten.

1/- post free.

**NURSING NOTES ON MIDWIFERY AND GYNÆCOLOGY.**

By Sister Ross, Rotunda Hospital. 1/1 post free.

**POCKET-BOOK TEMPERATURE CHARTS.**

17 Twelve-hour, 8 Four-hour Charts.
Perforated. 7d. post free.

### THE BURDETT SERIES.

***Small Nursing Text-Books.***

1/- each post free, or Eleven Volumes for 10/-.

**DISTRICT NURSING,**
**HOSPITAL AND PRIVATE NURSING,**
**MIDWIVES' POCKET-BOOK,**
**FEVERS AND INFECTIOUS DISEASES,**
**PHARMACY AND DISPENSING,**
**NURSING OF CHILDREN,**
**MENTAL NURSING,**
**SICK NURSING AT HOME,**
**GYNÆCOLOGICAL NURSING, and**
**SYLLABUS OF LECTURES TO NURSES.**

Price 1d. **THE NURSING MIRROR** Price 1d.

Published every Friday, and obtainable at this Office, and from all Booksellers and Newsagents on that day.

THE SCIENTIFIC PRESS, LTD.
28 & 29 Southampton Street, Strand, London, W.C.

